# Predictors of Marital Satisfaction Among Reproductive-Age Women Based on Personality Traits: A Study in Iran

**DOI:** 10.7759/cureus.59610

**Published:** 2024-05-03

**Authors:** Jamileh Abolghasemi, Fatemeh Jafari, Leila Neysani Samani, Shahnaz Rimaz

**Affiliations:** 1 Biostatistics, School of Public Health, Iran University of Medical Sciences, Tehran, IRN; 2 Midwifery, Mazandaran University of Medical Sciences, Sari, IRN; 3 Nursing Care Research Center, School of Nursing and Midwifery, Iran University of Medical Sciences, Tehran, IRN; 4 Radiation Biology Research Center, Iran University of Medical Sciences, Tehran, IRN

**Keywords:** agreeableness, conscientiousness, conflict, communication, personality, marital satisfaction

## Abstract

Background and objective: Marital satisfaction is a complex phenomenon influenced by a variety of factors, including personality traits, communication, conflict resolution, and economic stability. This study aims to examine the relationship between personality and marital satisfaction among reproductive-age women, exploring how these factors interplay and contribute to the overall dynamics of marital relationships.

Materials and methods: A cross-sectional study was conducted among reproductive-age women to assess marital satisfaction and personality traits using established assessment tools. The demographic data were collected through a structured questionnaire, and the relationship between personality traits and marital satisfaction was analyzed using confirmatory factor analysis (CFA). Regression models were used to identify significant predictors of marital satisfaction, and the fit of the CFA model was evaluated using various indicators.

Results: The mean age of the participants was 33.7 (±8.09) years, while the mean age of their husbands was 38.3 (±9.27) years. The results showed that conscientiousness and agreeableness had significant positive associations with marital satisfaction. Communication and conflict resolution emerged as key components of marital satisfaction, with standardized coefficients of 0.894 and 0.818, respectively. Financial management was also found to be strongly related to marital satisfaction, indicating the importance of economic stability in maintaining marital harmony.

Conclusion: The study underscores the significance of personality traits, communication, conflict resolution, and financial management in shaping marital satisfaction among reproductive-age women. The results suggest that interventions targeting improved communication and effective conflict-resolution strategies can enhance marital satisfaction. Additionally, addressing financial stressors and promoting economic stability could lead to better marital outcomes. These findings align with previous research, highlighting the need for a holistic approach to understanding and improving marital satisfaction. Further research is recommended to explore these relationships in different cultural contexts and with broader demographic groups.

## Introduction

Marital satisfaction serves as a fundamental pillar for healthy family dynamics, significantly influencing the emotional well-being of both partners [1]. Within the broader scope of public health, high levels of marital satisfaction are associated with improved mental health, reduced stress, and a more supportive environment for raising children [2,3]. For women of reproductive age, marital satisfaction is particularly important, as it directly impacts both their personal health and the overall health of their families. Research indicates that women experiencing higher marital satisfaction often have more favorable pregnancy outcomes and are better equipped to create nurturing environments for their children. This underscores the necessity of understanding the factors that contribute to marital satisfaction, paving the way for targeted interventions that support women during these critical life stages [4]. Personality traits have been extensively studied in relation to marital satisfaction, with research suggesting significant associations between certain personality characteristics and relationship outcomes. For example, studies have found that traits such as agreeableness, conscientiousness, and emotional stability are positively correlated with marital satisfaction, while traits like neuroticism and antagonism are often linked to lower levels of satisfaction [5]. By understanding how individual personality differences contribute to marital satisfaction among women of reproductive age, we can gain valuable insights into the mechanisms underlying relationship dynamics and develop targeted interventions to improve marital quality [6].

Demographic factors significantly shape marital satisfaction among couples. Variables such as age, age at marriage, duration of marriage, educational attainment, employment status, economic stability, divorce history, and substance addiction can all impact the quality of a marital relationship [7]. For instance, research indicates that couples who marry at a younger age or have shorter marriages may face unique challenges in navigating their relationships compared to those who marry later or have longer-lasting unions [8]. Similarly, socioeconomic factors like education and financial stability can influence communication, conflict resolution, and overall marital satisfaction [9]. A history of divorce or substance addiction in one or both partners can also introduce additional stress and conflict, possibly reducing satisfaction [10]. Understanding how these demographic factors affect marital satisfaction among women of reproductive age is crucial for developing tailored interventions and support services to address specific needs and challenges within these relationships. The aim of this study is to explore the predictors of marital satisfaction among reproductive-age women in Iran, with a specific emphasis on personality traits. By investigating the correlation between personality factors and marital satisfaction, while also considering the influence of demographic variables such as age, age at marriage, duration of marriage, educational attainment, occupation, economic status, history of divorce, and substance addiction, this research endeavors to shed light on the intricate relationship between individual characteristics and relationship outcomes. Ultimately, the study aims to enhance our understanding of the determinants of marital satisfaction among reproductive-age women in Iran, thereby informing the development of interventions and support services tailored to promote healthy and fulfilling marital relationships within this demographic.

## Materials and methods

This cross-sectional study was conducted on 400 reproductive-age women residing in Tehran. Inclusion criteria comprised married women of reproductive age who met the following conditions: absence of infertility inclination, absence of diseases that could jeopardize their own health or the fetus, chronic and untreatable physical and mental illnesses based on this study, absence of experiencing stress in the three months prior to sampling, and women within the age range of 15 to 49 years who have been married for at least one year and are simultaneously non-pregnant and non-lactating.

Sample size

To evaluate correlations at a 95% confidence level (α = 0.05) with an 80% test power (β = 0.20), and a correlation coefficient of 0.20 (which generally represents the largest sample size required when r = 0.2), the sample size was calculated using the following formula. Considering the cluster sampling design effect, a minimum sample size of 194 × 2 = 388 was required. In this study, we successfully collected 400 samples.

$n=\frac{\left ( Z_{1-\frac{\alpha }{2}}+Z_{1-\beta } \right )^{2}}{C^{2}}+3$ , $c=\frac{1}{2} ln\frac{1+r}{1-r}$ , $n= \frac{\left (1.96+0.84 \right )^{2}}{\left ( \frac{1}{2}ln\frac{1+0.2}{1-0.2} \right )^{2}}+3~\simeq 194$

In this study, we used data from 400 reproductive-age women residing in Tehran, selected through cluster sampling. Tehran's 22 districts were divided into five regions: north (three areas), south (five areas), east (five areas), west (five areas), and central (four areas). One area was randomly chosen from each region. In each selected area, three streets were chosen at random, and women meeting the study's inclusion criteria were identified. If they agreed to participate, they completed the questionnaires. For participants unable to complete the questionnaires due to illiteracy, we provided assistance in filling them out, ensuring adherence to ethical guidelines and avoiding any influence or bias in their responses.

Measures

This study employed three types of data collection instruments: a demographic survey, the ENRICH Marital Satisfaction Questionnaire, and the NEO Personality Inventory. The demographic section captured various details, including age (in years), age at marriage (in years), duration of marriage (in years), education level (categorized as illiterate, below diploma, diploma, or university), number of children, employment status (employed or unemployed), perceived family economic status (weak, moderate, or good), substance addiction (yes or no), and history of divorce (yes or no). The Enrich Marital Satisfaction Questionnaire consists of 47 items organized into nine components. In this study, we utilized the Persian version of the questionnaire [11,12]. The response spectrum utilizes a five-point Likert scale ranging from "strongly disagree" = 1 to "strongly agree" = 5. The components of the questionnaire are as follows: personality issues, communications, conflict resolution, financial management, leisure activities, sexual relationships, marriage and children, family and friends, and religious orientation. The NEO Personality Questionnaire consists of 60 items and covers five factors: conscientiousness, agreeableness, extraversion, openness to experience, and neuroticism [13,14]. Responses are given on a five-point Likert scale, ranging from "completely disagree" = 0 to "completely agree" = 4. Using data from 400 samples in this study, Cronbach's alpha values for the components of marital satisfaction ranged from 0.73 to 0.92, with an overall alpha of 0.87. For the personality components, Cronbach's alpha ranged from 0.76 to 0.87, with an overall alpha of 0.86. These values indicate that both the marital satisfaction and personality assessment tools exhibit good reliability, with the minimum acceptable Cronbach's alpha set at 0.7.

Statistical analysis

In this study, the normality of quantitative data was assessed using the Kolmogorov-Smirnov test. Descriptive statistics, including frequency, percentage, mean, and standard deviation (SD), were employed to analyze the general characteristics of the participants. Multiple linear regression with the forward method was used to identify demographic factors affecting marital satisfaction. To validate the structural model, confirmatory factor analysis (CFA) was performed on the components of marital satisfaction and personality. A significance level of P < 0.05 was considered for the tests (with P < 0.2 for single linear regression). Data analysis was carried out using R software (version 4.3.2).

## Results

The mean age of the women in this study was 33.7 years (±8.09), while the mean age of their spouses was 38.3 years (±9.27). An independent samples t-test showed a significant age difference between women and their husbands, with the women, on average, 4.6 years younger (p < 0.001). The distribution of educational levels among women and their spouses was similar, with no significant difference (p = 0.989). However, a chi-square test revealed a significant disparity in employment status between the two groups: 60% of women were employed, compared to 92% of men (p = 0.004). In terms of substance addiction, there was a notable difference, with men being about four times more likely to have an addiction compared to women (p < 0.001). Additionally, there was a significant difference in divorce history between women and their husbands, with men being approximately 1.5 times more likely to have a history of divorce (p < 0.001).

Table 1 summarizes the demographic information for the study participants and their husbands.

**Table 1:** Demographic Characteristics of Wives and Husbands in the Study SD, standard deviation

Variables	Wife	Husband
	Mean (SD)	Mean (SD)
Age	33.7 (8.09)	38.3 (9.27)
Age at Married	23.1 (4.7)	27.5 (4.82)
Duration of Marriage	10.9 (8.22)	
Number of Children	2.2 (1.01)	
	Number (%)	Number (%)
Education		
Illiterate	2 (0.5)	3 (0.7)
Below Diploma	22 (5.5)	22 (5.5)
Diploma	120 (30.0)	118 (29.6)
University	256 (64.0)	257(64.2)
Occupational Status		
Non-employee	236 (59.0)	32 (8.0)
Employee	164 (41.0)	368 (92.0)
Economic Status		
Weak	53 (13.3)	
Moderate	266 (66.5)	
Good	81 (20.2)	
Substance Addiction Status		
Yes	7 (1.8)	30 (7.9)
No	393 (98.2)	370 (92.1)
Divorce History		
Yes	16 (4.2)	23 (6.0)
No	384 (95.8)	377 (94.0)

In Table 2, we present the mean, SD, and percentage scores for each of the components in the marital satisfaction and personality assessment tools. To calculate the percentage score for the personality components, the mean score was divided by the number of component items, then by four, and the result was multiplied by 100. Similarly, to compute the percentage score for the components of marital satisfaction, the mean of each component was divided by its respective number of items, then by five, and the final result was multiplied by 100. As shown in Table 2, the overall percentage score for personality is 44.6%, while for marital satisfaction, it is 66%. These scores offer a quantitative representation of the general personality traits and marital satisfaction levels in our study's sample.

**Table 2:** Mean, SD, and % of Personality, Satisfaction, and Their Components Scores SD, standard deviation

	Component	No of Item	Mean	SD	%
Personality	Neuroticism	12	23.8	7.05	39.7
	Extraversion	12	26.9	5.92	44.8
	Openness	12	24.3	4.30	40.5
	Agreeableness	12	28.0	5.88	46.7
	Conscientiousness	12	31.0	7.32	51.7
	Personality Total	60	133.9	14.73	44.6
Marital Satisfaction	Personality Issues	6	12.6	3.74	52.5
	Communications	6	16.1	3.88	67.0
	Conflict Resolution	5	16.0	3.50	80.0
	Financial Management	5	12.7	3.90	63.5
	Leisure Activities	5	13.1	3.33	65.5
	Sexual Relationships	5	13.6	3.85	68.0
	Marriage and Children	5	13.0	3.23	65.0
	Family and Friends	5	14.8	2.66	74.0
	Religious Orientation	5	12.1	4.06	60.5
	Satisfaction Total	47	124.2	23.16	66.0

Linear regression model

We began by using single linear regression to identify factors affecting marital satisfaction, considering the demographic variables listed in Table 1. The significance level for this initial analysis was set at 0.2. All variables from Table 1, except education, showed significant correlations with marital satisfaction and were considered for inclusion in a multiple linear regression model. Using the forward method with a significance level of 0.05, we fitted the regression model, with the husband's age, husband's substance addiction, family's economic status, and women's divorce history emerging as significant variables in the final model. Table 3 summarizes the final results, displaying only the variables that were statistically significant in the multiple linear regression model. The multiple linear regression analysis showed that each additional year in the husband's age was associated with a 0.448-point increase in the women's marital satisfaction score. Women whose husbands were addicted to drugs had, on average, a 10-point lower marital satisfaction score. When compared to those with good economic status, women with poor and moderate economic statuses had, respectively, 16-point and five-point higher marital satisfaction scores. Additionally, women with a history of divorce had approximately 17 points lower marital satisfaction than those without a history of divorce.

**Table 3:** Results of the Multiple Linear Regression Model for Estimating Marital Satisfaction Among Study Participants (N = 400)

Variables	Coefficient	SD	P
Constant	94.812	5.626	<0.001
Husband Age	0.448	0.124	<0.001
Husband's Substance Addiction Status			
No	9.896	4.405	0.025
Yes	0		
Economic Status			
Weak	15.939	4.047	<0.001
Moderate	5.274	2.886	0.068
Good	0		
Divorce History of Wife			
No	17.236	5.620	0.002
Yes	0		

Confirmatory factor analysis

**Figure 1:**
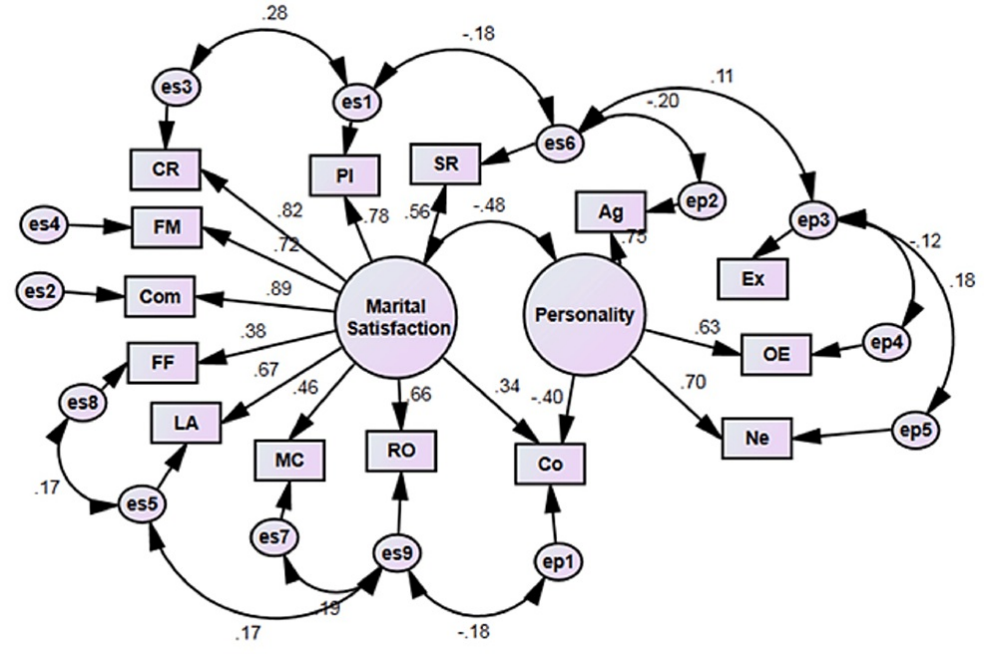
Fitted CFA Model Illustrating the Relationships Between Marital Satisfaction and Personality Components Among Reproductive-Age Women Marital satisfaction components include PI, Com, CR, FM, LA, SR, MC, FF, and RO. Personality components include Co, Ag, Ex, OE, and Ne PI, personality issues; COM, communications; CR, conflict resolution; FM, financial management; LA, leisure activities; SR, sexual relationships; MC, marriage and children; FF, family and friends; RO, religious orientation; CO, conscientiousness; AG, agreeableness; ex, extraversion; OE, openness to experience; NE, neuroticism; CFA, confirmatory factor analysis

In this study, we used CFA to examine the direct and indirect relationships between marital satisfaction, personality, and their components among women of reproductive age. The results of the CFA are presented in Figure 1. In this study, we employed CFA to explore the direct and indirect relationships between marital satisfaction, personality, and their components among women of reproductive age. The CFA results are depicted in Figure 1. As shown in Figure 1, the standardized regression coefficients between the various components of marital satisfaction and overall marital satisfaction range from 0.38 to 0.89, suggesting strong associations. Regarding personality variables, the correlations between four of the personality components and the overall personality construct range from 0.4 to 0.75. In our final fitted model, the extraversion component had no direct effect on the overall personality construct but exhibited indirect relationships through intermediary variables like openness to experience and neuroticism. Additionally, there was a weak negative correlation between openness to experience and extraversion.

The results in Table 4 provide an overview of the standardized and unstandardized regression coefficients derived from the CFA, focusing on the relationships between personality and marital satisfaction. For the personality factors, we found a negative relationship between conscientiousness and the overall personality construct. Specifically, the standardized coefficient for conscientiousness was -0.400, indicating that higher levels of conscientiousness are associated with lower personality scores. Conversely, agreeableness, openness to experience, and neuroticism demonstrated positive relationships with the personality construct. Among these, agreeableness had the strongest standardized coefficient at 0.750, suggesting a robust positive relationship. Openness to experience (0.629) and neuroticism (0.705) also showed significant positive correlations with the overall personality construct. When examining the relationship between the components of marital satisfaction and the overall satisfaction construct, communication emerged as the strongest factor, with a standardized coefficient of 0.894. This suggests that effective communication plays a crucial role in marital satisfaction. Other factors such as conflict resolution, financial management, leisure activities, personal issues, sexual relationships, marriage and children, family and friends, and religious orientation also showed positive relationships with marital satisfaction. The standardized coefficients for these factors ranged from 0.382 for family and friends to 0.818 for conflict resolution, indicating varying degrees of influence on marital satisfaction. Furthermore, the analysis revealed a positive relationship between conscientiousness and marital satisfaction, with a standardized coefficient of 0.340, suggesting that marital satisfaction can positively influence levels of conscientiousness. These findings highlight the complex and interconnected nature of personality and marital satisfaction. They suggest that different components of personality and marital satisfaction are intricately linked, with each factor potentially playing a unique role in contributing to the overall dynamics of marital relationships among women of reproductive age.

**Table 4:** Standardized and Unstandardized Regression Coefficients for Personality Traits and Marital Satisfaction Components in Women, Derived From the CFA Model CFA, confirmatory factor analysis

Variables			Estimate	SE	CR	P	Standardized
Conscientiousness	< - - -	Personality	-0.550	0.085	-6.494	<0.001	-0.400
Agreeableness	< - - -	Personality	0.859	0.078	10.971	<0.001	0.750
Openness to Experience	< - - -	Personality	0.719	0.071	10.152	<0.001	0.629
Neuroticism	< - - -	Personality	1.000				0.705
Leisure Activities	< - - -	Satisfaction	1.000				0.675
Financial Management	< - - -	Satisfaction	1.254	0.099	12.699	<0.001	0.722
Conflict Resolution	< - - -	Satisfaction	1.274	0.090	14.086	<0.001	0.818
Communications	< - - -	Satisfaction	1.543	0.102	15.136	<0.001	0.894
Personality Issues	< - - -	Satisfaction	1.298	0.096	13.462	<0.001	0.779
Sexual Relationships	< - - -	Satisfaction	0.962	0.095	10.168	<0.001	0.562
Marriage and Children	< - - -	Satisfaction	0.657	0.079	8.310	<0.001	0.457
Family and Friends	< - - -	Satisfaction	0.454	0.060	7.518	<0.001	0.382
Religious Orientation	< - - -	Satisfaction	1.191	0.092	12.923	<0.001	0.661
Conscientiousness	< - - -	Satisfaction	1.072	0.177	6.071	<0.001	0.340

Table 5 presents the model fit indices. Based on the indicators in Table 5, the CFA model used to explore the relationship between marital satisfaction and personality among reproductive-age women shows a good overall fit. The Chi-square minimum (CMIN)/df ratio and Root-mean-square error of approximation (RMSEA) values are within acceptable ranges, and the high comparative fit index (CFI) and normed fit index (NFI) scores indicate an excellent comparative fit. The Holter indices suggest moderate stability. These results suggest that the CFA model is appropriate and adequately reflects the relationships in this study.

**Table 5:** Indicators of the Fitted Model of CFA of Marital Satisfaction and Personality Among Reproductive-Age Women CMIN, Chi-square minimum; RMSEA, root-mean-square error of approximation; CFI, comparative fit index; NFI, normed fit index

	CMIN	df	CMIN/df	RMSEA	CFI	NFI	Holter 0.05	Holter 0.01
	138.487	66	2.098	0.054	0.996	0.991	374	412
Acceptable Range			2-3	<0.07	>0.9	>0.9	<400	>400

## Discussion

Table 1 provides key information about the demographic characteristics of the participants. The results indicate that the average age of the women in the study is 33.7 years, while the average age of their husbands is 38.3 years. This age difference suggests that husbands are typically slightly older when getting married, which aligns with the findings of Lee et al. [15]. Additionally, the mean age at marriage for women is 23.1 years and for men, it is 27.5 years, indicating that women generally marry at a younger age. The average duration of marriage is reported as 10.9 years, indicating that the study encompasses women with a relatively wide range of marital experiences, as noted in the findings of Entezari et al. [16]. The mean number of children is 2.2, suggesting that the families included in the study typically have a small number of children. In terms of educational attainment, the majority of women and their husbands hold a university degree or a high school diploma, indicating a relatively high level of education within this demographic. This level of education could contribute to a greater understanding of factors related to marital satisfaction. Employment status is another important demographic factor [7]. Most women in the study are not employed, while most husbands are employed. This difference in employment status could impact marital satisfaction, especially in cases where financial issues are concerned. Regarding economic status, most participants are in the moderate range, suggesting a fairly balanced distribution of economic conditions. However, a small percentage of participants report weak or good economic status, which could have distinct effects on marital satisfaction. In terms of drug use, a small percentage of both women and men in the study have issues with drug addiction [17]. Additionally, a small percentage of both women and men have a history of divorce, which could impact marital satisfaction and related factors [18]. These demographic findings provide a comprehensive picture of the participants' characteristics and allow for a deeper understanding of factors that might influence marital satisfaction.

Based on the results presented, the overall marital satisfaction percentage in our study is 66.0%, indicating a relatively high level of satisfaction among reproductive-age women. This value aligns with similar studies in other contexts, even though cultural, socio-economic, and other factors might affect these results [4]. Regarding personality traits, our study shows that conscientiousness has the highest mean score (51.7%), followed by agreeableness (46.7%). These findings are consistent with other studies that have indicated a positive correlation between conscientiousness and marital satisfaction [5]. Conscientiousness and agreeableness often correlate with more positive relationships and greater marital harmony. Concerning marital satisfaction components, our study indicates that conflict resolution, communication, and sexual relationships exhibit higher percentages. These findings are in line with other research demonstrating that effective communication and conflict resolution are key factors in maintaining marital satisfaction. Effective conflict resolution techniques and open communication between couples are among the most significant factors in successful marriages [19]. Although this study provides valuable data, it is crucial to consider its limitations. Cultural differences, changing social norms, and economic factors may influence the results [7]. Therefore, further research and comparison with various studies in different regions and communities are necessary to generalize the findings.

The observed negative correlation of -0.48 between personality and marital satisfaction in our CFA model indicates that specific personality traits, such as conscientiousness or neuroticism, might be linked to lower marital satisfaction. This finding aligns with previous research suggesting that personality traits, especially neuroticism, are associated with higher levels of conflict and reduced marital satisfaction [13]. Neuroticism, a component of the Big Five personality framework, is generally associated with negative emotions and increased emotional volatility, which can contribute to conflicts within marital relationships. Similarly, while conscientiousness is often connected to responsible and organized behavior, excessive levels of this trait may lead to rigidity and inflexibility, negatively impacting marital satisfaction. Many studies, such as those by Karney and Bradbury, have shown that personality traits can significantly influence the quality and longevity of marital relationships [8]. The negative correlation between personality and marital satisfaction in this CFA model suggests that targeted strategies to enhance positive personality traits while minimizing negative ones could lead to improved marital satisfaction.

The regression coefficients obtained from the CFA in Table 4 provide critical insights into the relationships between personality traits, marital satisfaction, and their components. These findings offer an opportunity to discuss the implications for reproductive-age women in the context of marital satisfaction. One of the significant results from the CFA is the relationship between personality and conscientiousness, with a standardized regression coefficient of -0.400 (p < 0.001). This negative association may indicate that individuals with higher conscientiousness are less likely to exhibit extreme personality traits, suggesting a more stable disposition in marital relationships. Research by Roberts et al. suggests that conscientiousness is associated with reliability and discipline, qualities that can lead to higher marital satisfaction [20]. On the other hand, the positive correlation between personality and agreeableness (standardized coefficient of 0.750, p < 0.001) underscores the importance of a cooperative and compassionate demeanor in marital relationships. Agreeableness is associated with qualities such as empathy and kindness, which contribute to healthier relationships and conflict resolution [21]. Regarding marital satisfaction, the CFA results demonstrate that communication (standardized coefficient of 0.894, p < 0.001) has the strongest relationship with satisfaction. This finding aligns with Gottman's research, which emphasizes that effective communication is one of the key predictors of successful marriages [22]. Similarly, Conflict resolution also exhibits a strong relationship with satisfaction (standardized coefficient of 0.818, p < 0.001), further supporting the idea that couples who can effectively resolve conflicts tend to report higher marital satisfaction [23]. Another important observation is the relationship between financial management and satisfaction, with a standardized coefficient of 0.722 (p < 0.001). This finding indicates that economic stability plays a crucial role in marital satisfaction. Studies have shown that financial stress can lead to increased marital discord, suggesting that effective financial management is vital for maintaining marital harmony [24]. In summary, the CFA results provide a nuanced view of the relationships between personality traits, marital satisfaction, and their components. These findings are consistent with previous research, indicating that qualities such as agreeableness and effective communication play crucial roles in promoting marital satisfaction among reproductive-age women. The results also suggest that addressing financial management and conflict resolution strategies can further enhance marital satisfaction.

Limitation

There were no specific limitations identified in this study.

## Conclusions

This study explored the relationship between personality traits and marital satisfaction among reproductive-age women. The findings revealed that the overall marital satisfaction score was 66.0%, indicating a moderate to high level of satisfaction. However, a negative correlation between certain personality traits and marital satisfaction suggests that specific personality factors, like high levels of conscientiousness, might be associated with lower marital satisfaction. Conscientiousness and agreeableness emerged as the most strongly correlated personality traits with marital satisfaction, underlining their role in fostering positive relationships. Key factors contributing to marital satisfaction included effective communication, conflict resolution, and financial stability, with communication being the most significant predictor of satisfaction. The study's results suggest that enhancing communication and conflict resolution skills, along with addressing financial stress, could positively influence marital satisfaction. Future research could explore the specific roles of personality traits in marital relationships while considering various cultural factors to further enrich the understanding of these dynamics.
